# The Burden of Suicide in Rural Bangladesh: Magnitude and Risk Factors

**DOI:** 10.3390/ijerph14091032

**Published:** 2017-09-09

**Authors:** Shumona Sharmin Salam, Olakunle Alonge, Md Irteja Islam, Dewan Md Emdadul Hoque, Shirin Wadhwaniya, Md Kamran Ul Baset, Saidur Rahman Mashreky, Shams El Arifeen

**Affiliations:** 1 International Centre for Diarrhoeal Disease Research, GPO Box 128, Dhaka 1000, Bangladesh; irteja.islam@icddrb.org (M.I.I.); emdad@icddrb.org (D.M.E.H.); shams@icddrb.org (S.E.A.); 2 Department of International Health, Johns Hopkins University Bloomberg School of Public Health, Baltimore, MA 21205, USA; oalonge1@jhu.edu (O.A.); swadhwa2@jhu.edu (S.W.); 3 Center for Injury Prevention and Research, House # B-162, Road # 23, New DOHS, Mohakhali, Dhaka 1206, Bangladesh; kamran_baset@yahoo.co.uk (M.K.U.B.); mashreky@ciprb.org (S.R.M.)

**Keywords:** suicide, attempted suicide, burden, risk factors, rural, Bangladesh, injury, violence

## Abstract

The aim of the paper is to quantify the burden and risk factors of fatal and non-fatal suicidal behaviors in rural Bangladesh. A census was carried out in seven sub-districts encompassing 1.16 million people. Face-to-face interviews were conducted at the household level. Descriptive analyses were done to quantify the burden and Poisson regression was run to determine on risk factors. The estimated rates of fatal and non-fatal suicide were 3.29 and 9.86 per 100,000 person years (PY) observed, respectively. The risk of suicide was significantly higher by 6.31 times among 15–17 and 4.04 times among 18–24 olds compared to 25–64 years old. Married adolescents were 22 times more likely to commit suicide compared to never-married people. Compared to Chandpur/Comilla district, the risk of suicide was significantly higher in Narshingdi. Students had significantly lower risk of non-fatal suicidal behavior compared to skilled laborers. The risk of non-fatal suicidal behavior was lower in Sherpur compared to Chandpur/Comilla. Among adolescents, unskilled laborers were 16 times more likely to attempt suicide than students. The common methods for fatal and non-fatal suicidal behaviors were hanging and poisoning. Suicide is a major public health problem in Bangladesh that needs to be addressed with targeted interventions.

## 1. Introduction

According to the 2014 WHO Global Health Estimates, there were about 803,900 suicides in 2012 representing 1.4% of the global burden of disease or over 39 million disability adjusted life years (DALYs) lost [1]. Fatal suicidal behavior or suicide is death resulting from self-harm. Worldwide, suicide accounted for 16% of injury mortality and 1.4% of total mortality in 2012, making it the 15th most common cause of death for all age groups in that year [1,2]. Analysis of trends indicate that the overall suicide rate has decreased significantly over the past decade. The global suicide rate was 11.4 per 100,000 population in 2012, a decrease from 14.4 per 100,000 in 2000 [1]. However, the rate of decline flat-lined in the latter part of that decade, and the rate in 2008 (11.6 per 100,000) was about the same as rate reported for 2012 [3,4,5]. Globally, suicide remains the second leading cause of death among 15–29 year olds [1,2]. In addition to these striking facts for suicide, there are indications that, for every adult that dies by suicide, there are 10–20 more who attempted a suicide event [2,5]. This assumption for suicide attempts is based on the WHO world mental health surveys among adults (>18 years of age) in 21 low-middle and high-income countries who report a prevalence of 3–4 per 1000 individuals [6].

Fatal and non-fatal suicidal behaviors have been found be mostly prevalent among the vulnerable populations of the world where availability of and accessibility to resources and services for identification and treatment are scarce [2]. In fact, three-fourths of the global suicides have been estimated to occur in low and middle-income countries (LMICs) [2]. The southeast Asia region alone accounts for 40% of the global suicide deaths, with China and India being the leading contributors [2,4]. Although there are variations in trends and risk factors of suicide among countries in the southeast Asia region, compared to Western countries, the region as whole has higher suicide rates, lower gender (male-to-female) suicide rate gap, higher rates among the elderly, and an increasing trend of youth suicides [5,7]. Reviews and epidemiological data also indicate that there exists wide urban-rural disparity in suicide, with suicide rates often greater in rural areas [5,8,9].

Like most southeast Asian countries, a fundamental challenge for Bangladesh is the lack of quality suicide data or system for monitoring and surveillance. In Bangladesh, a limited number of studies have attempted to quantify suicide rates. Mashreky et al. suggested that about 10,000 people commit suicide in the country in a year [10], and the rate of suicide was found to be 7.3 per 100,000 population (6.5 in males and 8.2 in females) and was highest in the 60+ age group and considerably high among adolescents [10]. These estimates for were based on the 2003 Bangladesh Health and Injury Survey [10]. According to WHO Global Health Estimates, the suicide rate for 2012 in Bangladesh was 7.8 per 100,000 population (8.7 in females and 6.8 in males) [11]. Demographic and health surveillance in two rural sub-districts of Bangladesh between 2004 and 2010 revealed that the most common cause of death for young adults (aged 15–49) was injury (23.5%) with suicide accounting for 11.9% [12]. Other studies that have attempted to determine the causes of female deaths, revealed a growing rate of suicide among adolescent females [13]. Analysis of results from national household surveys in 2001 and 2010 and hospital based surveys in 1996–1997 suggested that suicide was the main cause for deaths among adolescent females, accounting for 16–22% of all deaths in this age group [13]. There is a need for recent population-based data that could be used to define the burden and epidemiology of suicide in Bangladesh, and to consequently address interventions for reducing rates of fatal and non-fatal suicidal behavior in Bangladesh.

The objective of this paper is to quantify the burden and socio-demographic risk factors of fatal and non-fatal suicidal behaviors in rural Bangladesh. The goal is to identify high-risk subgroups, demonstrate any transition in causes of death in the country and to call for action on the need to recognize and address this enormous public health issue on a national scale. It is hoped that this paper will provide evidence to influence national policies to address and invest in related research and preventive approaches.

## 2. Materials and Methods 

### 2.1. Study Design, Area and Population

The data for this paper is from an implementation research study conducted with an aim to reduce drowning in under-five children in five districts of rural Bangladesh [14,15]. A cross-sectional baseline census was conducted over a six months period from June through November 2013 before the start of the implementation of the drowning prevention interventions [16]. The census covered 1.165 million people in 51 unions from seven sub-districts of Bangladesh (and this study describing the epidemiology of fatal and nonfatal suicide behaviors is based on the census). The sub-districts included were Matlab North, Matlab South, Daudkandi, Chandpur Sadar, and Manohardi in the central section of the country, and Raiganj and Sherpur in the north.

### 2.2. Questionnaire and Data Collection

The baseline census collected information on fatal and non-fatal injury outcomes including fatal and non-fatal suicidal behaviors, characteristics of the underlying injury mechanisms and health seeking behavior for the injury or death outcome on all populations in the census area. In addition, the census collected information on social and demographic characteristics, physical environment and birth history. This was done using a questionnaire covering seven modules (I. Household characteristics and socioeconomic census; II. Birth history; III. Household environment, IV. Death confirmation; V. Injury morbidity; VI. Injury mortality and VII. Injury mechanisms). The injury mechanisms module (module VII) comprised of 12 forms covering 12 injury events, the first of which was attempted suicide. In addition, there were three notification forms: injury notification, death notification and child notification, which provided notifications of any injuries or deaths including suicide and attempted suicide from module I. All fatal injury information (including fatal suicidal behavior) was collected over a one-year recall period, whereas non-fatal injuries (including non-fatal suicidal behavior) were collected over a six-month recall period. 

Suicide (or non-fatal suicidal behavior) was operationally defined as a self-initiated injury event that resulted in a fatal outcome (or non-fatal outcome). The injury events assessed were defined based on ICD-10 classification, and are described in details elsewhere [16].

All information was collected directly from the head of households or any adults 18 years above with sufficient knowledge of the household. All forms were written in English and translated to Bangla. The forms were back-translated and pilot-tested prior to the actual data collection.

Data collection was implemented by two sets of trained data collectors. The first set of data collectors completed the questionnaire forms in module I–III and notified of any fatal suicide or non-fatal suicide events. In module I after enlisting all the household members and their soico-demographic information, we asked “whether the member had suffered from any non-fatal injuries (read out all injuries) in the past six months”. In order to obtain information on deaths we asked “whether there were any deaths in the past one year”. Based on the notification, a second set of data collectors completed the questionnaire forms in module IV–VII only in households with the suicide injury or death event. Each set of the questionnaire forms took between 40–50 min to complete. Written informed consent was obtained prior to data collection. 

### 2.3. Statistical Method and Analyses

Descriptive statistics were used to describe the population by various variables such as age, gender, marital status, educational attainment, wealth quintile and geographical area. The overall rates of suicide and attempted suicide was calculated per 100,000 person years observed (PYO). The mortality and morbidity rates were also stratified by the above-mentioned variables. Principal component analysis was done to estimate the wealth quintile using various variables that indicate asset availability and housing conditions. 

Multivariable analysis was performed with multilevel Poisson regression models. Model estimates were reported as incidence rate ratios (IRR), with their respective 95% confidence intervals. The models were first implemented with one covariate, and then adjusted for other covariates and possible confounders (age, gender, marital status, educational attainment, wealth quintile and geographical area). Age, education and occupation were considered as categorical variables, whereas gender was considered as a binary predictor (male as reference group). Socioeconomic quintile was considered as ordinal data from lowest to highest. Age was theorized to modify the risk of fatal and non-fatal suicidal behavior with regards to other variables, especially marital status in rural Bangladesh. Thus, separate analyses were implemented for (1) individuals 15 years and older, and (2) adolescents-only (aged 10–17 years) to adjust for the possible interactions with age. The final results for individuals above 15 years of age are presented in the paper, and results for the adolescent-only analyses have been included in the (Supplementary Materials Tables S1 and S2). 

Variable construction and estimations were done with the statistical software STATA 13 (Stata Corp. 2013. Stata Statistical Software: Release 13. College Station, TX, USA: Stata Corp LP.). 

### 2.4. Ethical Statement

Ethical approval was obtained from of the Institutional Review Board of the Johns Hopkins Bloomberg School of Public Health (JHSPH) (ethics approval code is 00004746); and Ethics Review Committees of International Centre for Diarrhoeal Disease Research, Bangladesh (icddr,b) and Center for Injury Prevention and Research Bangladesh (CIPRB) under the study Saving of Lives from Drowning (SoLiD), Bangladesh.

## 3. Results

The survey covered a population of 1,169,593 (Table 1). Overall, 21.6% of the population was less than 10 years of age, 72.3% were between 10–65 years of age and only 6.1% of the population was above 65 years. More than three-fifth (65%) of the respondents had received at least primary education and about half (48.8%) of the population were married during the time of the survey. Three-fourths (74.6%) of the population were not employed and were either retired, unemployed, housewives, students or children. Among those who were employed, a majority were involved in agricultural activities (9%), followed by skilled work (7.6%) and business (5.3%). Household wealth quintiles were evenly distributed. The majority of respondents resided in Matlab North (22.7), Sherpur (19.5%), Matlab South (17.9%) and Manohardi (17.5%) sub-districts of the country.

A total of 38 fatal suicide and 57 non-fatal suicidal events were recorded in the past one year and six months prior to the survey, respectively (Table 2). In rural Bangladesh, the estimated rates of fatal and non-fatal suicidal behavior were 3.29 per 100,000 PYO and 9.86 per 100,000 PYO. Suicide rates were found to be higher among the younger population specifically the adolescents compared to the adults and the elderly. The rates were highest among 15–17 year olds (11.3 per 100,000 PYO), followed by 18–24 year olds (7.5 per 100,000 PYO) and 3.52 per 100,000 PYO among young adolescents (aged between 10–14 years old). There was no difference in the rates of fatal suicidal behavior comparing males and females of all ages, and the gender ratio (male-to-female) was 1.18. The rates did not vary much by marital status and no definitive pattern in suicidal behavior was observed by education or wealth quintile. The rate was highest among those who had completed 10 years of education or had no education, and among those belonging to the middle wealth quintile. The suicide rate was found to be high among the transport workers (17.66 per 100,000 PYO), but was similar among other occupations, ranging from 3.38 in skilled labors to 4.80 in those involved in agricultural activities. Geographically, suicide rates were found to be higher in Raiganj and Manohardi Sub-districts in Northern Bangladesh compared to the sub-districts of Matlab situated in central Bangladesh. 

Table 3 presents stratified analysis of suicide rates by age and sex. Among males, suicide rates are highest among the 15–17 year olds followed by 10–14 year olds, whereas among females the rates are highest among the 15–17 year olds and 18–24 year olds. The rates of suicide are higher in females among 15–24 year olds than in males of the same age group. The rates are, however, higher in males for 10–14 and 25–64 year olds.

In contrast to suicide rates, the rates of non-fatal suicidal behavior were highest among individuals aged 18–24 year olds (19.53 per 100,000 PYO), 25–64 year olds (13.46 per 100,000 PYO) followed by 15–17 year olds (12.92 per 100,000 PYO). The rates were highest in people involved in un-skilled labor (32.87 per 100,000 PYO) and agricultural activities (21.23 per 100,000 PYO) and more common in people belonging to the bottom three wealth quintiles. Non-fatal suicidal behavior was also evident among women who were widowed, divorced or separated (10.45 per 100,000 PYO), which was not present in the case of suicide. Along with Raiganj (21.31 per 100,000 PYO) and Manohardi (11.88 per 100,000 PYO) sub-districts, the rates were also high in Matlab South (13.53 per 100,000 PYO).

Table 4 presents crude and adjusted IRR for fatal suicide and non-fatal suicidal behavior among respondents aged 15 years or more in the study. The significant factors associated with fatal suicidal behavior in the adjusted model included age, and geographical region (Table 4). The adjusted risk of fatal suicidal behavior was clearly higher, by six and four times among the 15–17 year olds (IRR 6.31, CI 1.35–29.32) and 18–24 year olds (IRR 4.04, CI 1.56–10.47) respectively, compared to those aged between 25–64 years old. In addition, the risk was significantly higher in the Narshingdi district (IRR 2.89, CI 1.26–6.65) compared to Chandpur/Comilla district. Although transport workers were more likely to commit suicide compared to skilled labor, the relationship was not significant in the adjusted model. In addition, adjusted analysis only among adolescents aged between 10–17 year olds revealed that the risk of fatal suicidal behavior was 22 times higher among those who were married compared to those who were never-married (IRR 22.06, CI 3.70–131.63) (Supplementary Materials Table S1). 

The two factors that were significantly associated with non-fatal suicidal behavior in both the adjusted model among respondents aged 15 years or older included occupation and districts (Table 5). Compared to those involved in skilled labor, students were found to have a significantly lower risk (adjusted) of non-fatal suicidal behavior (IRR 0.21, CI 0.04–1.00). People living in Sherpur were found to be at significantly lower risk compared to those living in Chandpur/Comilla sub-districts (IRR 0.28, CI 0.08–0.94). Although, the risk of non-fatal suicidal behavior was twice among people living in Sirajganj district compared to those living in Chandpur/Comilla districts (IRR 2.10, CI 1.01–4.36) in the unadjusted model, the relationship was not significant in the adjusted model. Among adolescents aged 10–17 years, the risk of non-fatal suicidal behavior (adjusted) was 16 times higher among those involved in unskilled labor compared to students (IRR 15.75. CI 1.35–184.52) (Supplementary Materials Table S2).

The most common method of suicide was hanging (59%) followed by poisoning (31%), burn (5.1%), drowning (2.6%) and exsanguination (2.6%). These frequencies were reversed for non-fatal suicidal behavior, where poisoning (71.93%) was the most common method followed by hanging (22.81%), implying that the survival rate was higher among those who attempted to use poisoning as the method. Pesticides were the most commonly used poisoning material (62.7% in case of non-fatal suicidal behavior and 83% in case of fatal suicidal behavior). About two-thirds of the cases undertook the events at home followed by in-laws and other’s house (about one-fifth) and only a small proportion of cases chose public places for the event. There were no differences in the methods and place by gender and the pattern was similar.

## 4. Discussion

This paper set out to explore the epidemiology of fatal and non-fatal suicidal behavior in rural Bangladesh. The estimated rates of fatal and non-fatal suicidal behavior were 3.29 and 9.86 per 100,000 PYO, respectively. The significant factors associated with fatal suicidal behavior included age, marital status and geographical region, whereas the two factors that were significantly associated with non-fatal suicidal behavior were occupation and districts. 

The estimated rate for fatal suicidal behavior was 3.29 per 100,000 PYO (3.25 per 100,000 population per year), whereas the rate for non-fatal suicidal behavior was 9.86 per 100,000 PYO (4.87 per 100,000 population per six months) indicating that approximately 5000 people lost their lives to suicide and another 15,000 attempted a non-fatal suicide event in 2013. This rate for fatal suicidal behavior was, however, two times lower than the overall rate presented in the Global Health Estimates for 2012 [1] and almost four times lower than the rate of suicide in rural Bangladesh reported by Mashreky et al. [10]. Perhaps, this may be because the current data is only representative of rural Bangladesh, and significant differences may exist in suicide rates between urban and rural populations in Bangladesh. In addition, the rates are lower than that of other Asian countries such as China (9.8) [8], India (22.0) [9], South Korea (31.0), Japan (24.0), Sri Lanka (23.0), Taiwan (17.6), and Hong Kong (13.8) [5,8]. The rural rates in several of these Asian countries, including China, India, Sri Lanka, Japan, and Taiwan have been found to be higher compared to urban rates [5,8,9]. Disaggregation by gender and age reveals further disparity in urban-rural rates in these countries. Several contextual factors have been described to contribute to the increased risk of suicide in rural areas, including socio-economic and cultural differences, availability and accessibility to services, access to means, and, community attitudes towards mental illness and care-seeking [17,18,19] The rates presented for different countries should be interpreted with caution since only a few countries in the world have good vital registration or data collection systems that help obtain high quality data. For example, estimates in countries such as Hong Kong, Japan, Malaysia, Singapore, South Korea, and Taiwan are considered to be more reliable compared to countries such as China, India, Thailand and Sri Lanka [5]. The situation is further complicated by issues such as stigmatization and illegality that result in misclassification and under-reporting of suicide and suicidal attempts. It must be noted that Bangladesh is predominantly a Muslim country with not only strong religious sanctions against suicide and suicidal attempts but also has punitive laws against attempted suicide (Bangladesh penal code, 1860).

The gender gap (male to female ratio) for suicide was very low at 1.2. The findings contrast with High Income Countries (HIC), where suicide rates for males are about 3–4 times higher than those for females but similar to other Low and Middle Income Countries (LMICs) where the gender gap is low (1.6) [2,4,20,21]. Several Asian countries such as China, Hong Kong, Japan, India, Taiwan, Singapore, and Sri Lanka also have a lower gender ratio although there is some evidence that the gap may be increasing [5,7,8,22,23]. There are many potential reasons for different suicide rates in men and women: gender equality issues, differences in socially acceptable methods of dealing with stress and conflict for men and women, availability of and preference for different means of suicide, availability and patterns of alcohol consumption, and differences in care-seeking rates for mental disorders between men and women.

Our study also revealed that risks of fatal and non-fatal suicidal behavior were higher among the young, including the adolescents especially those 15–17 year olds, and young adults 18–24 year olds. The rates of suicide varied by age and sex with female 15–24 year olds individuals to be more likely to commit suicide compared to their male counterparts. Several studies in Bangladesh have also attempted to highlight this issue indicating that the young people, and not the elderly, are particularly vulnerable to injuries including self-harm and suicide in the country [12,13,24,25,26,27]. Despite this, there has been no study in Bangladesh that has aimed to determine the factors influencing high suicide rates among adolescents and young adults.

We conducted separate regression analysis among adolescents age 10–17 years and found early marriage to significant stressor for suicide, which explains why the suicide burden affect the female adolescents more. Such high suicide rates among adolescent women may be because of early and forced marriage, marital abuse related to dowry, and complete lack of opportunity for advancement and development coupled with the tendency to impulsive behavior and actions that is typical of young adolescents. Several studies, particularly in the West and developed regions, indicate that marriage is protective [28,29,30,31], while others in many developing countries indicate that it is a significant source of abuse and stress, particularly for the women, leading to higher psychiatric morbidity, suicidal ideation and suicide [9,32,33,34,35,36,37,38,39]. Our study did not find marriage to be protective of suicide for women of all ages. In addition, we found non-fatal suicidal behavior rates to be higher in adolescents who were involved in unskilled labor compared to those who were students. Psychological, familial, social, and cultural factors risk factors that have been identified to influence adolescents to commit suicide include low socio-economic and educational status, family history of suicide, parental separation, divorce, or death, poor relationship with family and peers, social contagion, prevalence of psychiatric disorder, psychological stressors, sexual abuse, substance abuse, social deprivation, and availability of high lethality methods (e.g., guns) [27,40,41,42]. Another emerging issue is the media representation of suicidal behavior including the modern internet that have been found to have substantial influence on the vulnerable including adolescents in some Asian countries [40,43]. 

Although not significant, the rates of fatal and non-fatal suicidal behavior were very high among agricultural workers and unskilled labor. Compared to skilled laborers students were, however, found to have lower rates of non-fatal suicidal behavior. This needs further attention and research since several studies have indicated that acute life stressors and poor educational status are significantly associated with higher rates of fatal and non-fatal suicidal behavior [5,21,44,45]. A variation was also seen geographically where fatal and non-fatal suicidal rates were found to be more in Sirajganj and Narsingdi districts of the country indicating the need to provide attention to particular socio-demographic or other factors in the regions. 

Studies reporting methods of suicide indicate that availability of and accessibility to methods influence the choice of the method and act as factor in increasing overall fatal and non-fatal suicidal events [46]. As such, the common suicide methods not only vary by age and sex but also shift with the availability/restriction of new methods and technologies [47]. Globally, ingestion of pesticide, hanging and firearms are among the most common methods of suicide, whereas poisoning and hanging has been found to be most common in Asia [2,5,7,47,48]. Our study findings are consistent with those in Asia and reports hanging followed by poisoning to be the most common methods for suicide. For non-fatal suicidal behavior, the most common method was poisoning followed by hanging. Rural economy in Bangladesh is predominantly agricultural where availability of pesticides is rampant and unregulated. Several studies have indicated that, despite some challenges, means restriction through legal regulations, safe storage, awareness could be an important strategy for controlling fatal and non-fatal suicidal behavior at the population level [43,46,49,50]. For example, restrictions on the import and sales of pesticides in Sri Lanka resulted in reductions in suicide in both men and women of all ages with 19,769 fewer suicides occurring in 1996–2005 compared to 1986–1995 [51]. In addition to this research on suicide prevention also indicates the need for national strategy and community based approaches focusing on targeted groups [52,53]. The application of findings of prevention studies from other countries should be interpreted with caution given the unique sociocultural context of Bangladesh, and implementation research on suicide prevention in the country is required.

### Limitations and Challenges

The study was conducted in rural Bangladesh and might not be generalizable for the country as risk factors might be very different in urban Bangladesh. Our study findings are generalizable to rural Bangladesh and similar settings in other low and middle income countries The fatal and non-fatal suicide rates, especially for non-fatal suicidal behavior, may be underreported due to challenges in data collection, recall bias, stigmatization and for punitive reasons. Given that information obtained from the head of the households may be subject to recall and social desirability biases, and to avoid stigma and likely legal punishments, suicide (especially of children and adolescent females) may be underreported, leading to biased fatal and nonfatal suicide rates. Contemporaneous and real-time death review/social autopsy may provide less biased estimates. Although mental illness is widely accepted as a risk factor of fatal and non-fatal suicidal behavior, our study was not designed to elicit any information on mental illness.

## 5. Conclusions 

This study has made an attempt to provide updated information on the burden of suicide and suicidal attempts from a population census that covered almost 1.2 million rural people in five districts of the country. We conclude that suicide is a serious public health problem in Bangladesh especially among the high-risk individuals such as adolescent women and married women; some of the factors associated with completed and attempted suicide are similar or vary compared to other Asian and LMIC countries. Considering the context, where resources and services for suicide identification, treatment and support are limited, there is a need to develop targeted national strategies and action plans, prevention programs and conduct further research to learn more about the constraints and reduce the rate of suicide and its associated risk factors. Strategies to uplift the status of women in rural Bangladesh, such as creating opportunities that would improve financial independence, programs to create awareness of mental health issues and access to mental health treatment among married adolescent women, are among a few potential paths to take to address the huge public health burden of suicide. Further research to adequately characterize the burden of suicide and the gender disparities in suicide and attempted suicide rates in the country are needed. Implementation research on community-based prevention strategies, such as counseling/support groups and mental health awareness, is also needed to support the development of a national strategy.

There is also an urgent need to establish reliable systems to continuously collect data on suicide in Bangladesh that will help interested agencies to measure the social and economic burden needed to drive the establishment and implementation of effective suicide prevention programs in the country.

## Figures and Tables

**Table 1:** Socio-demographic characteristics of the respondents.

Characteristics	*N* = 1,169,593	%
Age (in years)		
<10	252,392	21.6
10–14	142,121	12.2
15–17	62,098	5.3
18–24	133,534	11.4
25–64	508,059	43.4
65+	71,389	6.1
Sex		
Male	567,674	48.5
Female	601,919	51.5
Education ^1^		
No education	295,314	25.3
Primary complete (five years)	407,923	34.9
Secondary complete (10 years)	289,658	24.8
Secondary and above	63,873	5.5
Under five children	112,664	9.6
Occupation ^2^		
Agriculture	104,956	9.0
Business	61,661	5.3
Skilled labor	89,151	7.6
Unskilled labor	24,520	2.1
Transport worker	17,037	1.5
Students	312,537	26.7
Retired/unemployed/housewife	408,583	35.0
Children	144,454	12.4
Others (NA)	5948	0.5
Marital Status		
Married (Reference)	571,206	48.8
Never-married	227,319	19.4
Widowed/Divorced/Separated	59,033	5.0
Children (<12 years)	312,035	26.7
Wealth quintile		
Lowest	211,601	18.1
Second	218,695	18.7
Middle	238,371	20.4
Fourth	247,716	21.2
Highest	253,210	21.6
Sub-district		
Matlab North	265,897	22.7
Matlab South	209,772	17.9
Chandpur Sadar	128,356	11.0
Raiganj	104,357	8.9
Sherpur	228,519	19.5
Manohardi	204,319	17.5
Daudkandi	28,373	2.4
District		
Chandpur/Comilla	632,398	54.1
Sirajganj	104,357	8.9
Sherpur	228,519	19.5
Narshingdi	204,319	17.5

^1^ Information on education missing for 0.01 (161) participants; ^2^ Information on occupation missing for 0.06 (746) participants.

**Table 2:** Distribution of suicide and attempted suicide rates by socio-demographic and geographical factors, rural Bangladesh.

Characteristics	*N*	Fatal Suicidal Behavior			Non-Fatal Suicidal Behavior		
		Person Years Observed (PYO)	Frequency	Mortality Rate per 100,000 Person Years Observed (PYO)	Person Years Observed (PYO)	Frequency	Morbidity Rate per 100,000 Person Years Observed (PYO)
Total	1,169,593	1,153,901	38	3.29	578,046	57	9.86
Age (in years)							
<10	252,392	240,739	0	0.00	122,966	0	0.00
10–14	142,121	141,849	5	3.52	70,894	4	5.64
15–17	62,098	61,972	7	11.30	30,965	4	12.92
18–24	133,534	133,247	10	7.50	66,568	13	19.53
25–64	508,059	506,348	14	2.76	252,581	34	13.46
65+	71,389	69,745	2	2.87	34,072	2	5.87
Sex							
Male	567,674	559,566	20	3.57	280,538	28	9.98
Female	601,919	594,335	18	3.03	297,508	29	9.75
Education ^1^							
No education	295,314	293,176	11	3.75	145,672	16	10.98
Primary complete (five years)	407,923	406,727	9	2.21	203,011	17	8.37
Secondary complete (10 years)	289,658	288,858	17	5.89	144,209	22	15.26
Secondary and above	63,873	63,690	1	1.57	31,787	2	6.29
Under five children	112,664	101,294	0	0.00	53,291	0	0.00
Occupation ^2^							
Agriculture	104,956	104,221	5	4.80	51,802	11	21.23
Business	61,661	61,397	0	0.00	30,600	1	3.27
Skilled labor	89,151	88,864	3	3.38	44,330	8	18.05
Unskilled labor	24,520	24,417	1	4.10	12,169	4	32.87
Transport worker	17,037	16,989	3	17.66	8480	1	11.79
Students	312,537	311,935	12	3.85	155,892	6	3.85
Retired/Unemployed/Housewife	408,583	406,478	14	3.44	202,394	26	12.85
Children (Under 12 years)	144,454	133,003	0	0.00	69,125	0	0.00
Others (NA)	5948	5857	0	0.00	2886	0	0.00
Marital Status							
Married	571,206	568,601	25	4.40	283,292	37	13.06
Never-married	227,319	226,802	11	4.85	113,320	15	13.24
Widowed/Divorced/Separated	59,033	58,192	0	0.00	28,705	3	10.45
Children (Under 12 years)	312,035	300,306	2	0.67	152,729	2	1.31
Wealth quintile							
Lowest	211,601	208,601	7	3.36	104,479	10	9.57
Second	218,695	215,949	5	2.32	108,190	13	12.02
Middle	238,371	235,239	10	4.25	117,830	14	11.88
Fourth	247,716	244,482	9	3.68	122,460	11	8.98
Highest	253,210	249,630	7	2.80	125,088	9	7.19
Sub-district							
Matlab North	265,897	262,510	7	2.67	131,406	11	8.37
Matlab South	209,772	206,600	5	2.42	103,492	14	13.53
Chandpur Sadar	128,356	126,659	3	2.37	63,475	5	7.88
Raiganj	104,357	103,052	5	4.85	51,612	11	21.31
Sherpur	228,519	225,476	6	2.66	113,055	4	3.54
Manohardi	204,319	201,641	12	5.95	101,004	12	11.88
Daudkandi	28,373	27,962	0	0.00	14,002	0	0.00
District							
Chandpur/Comilla	632,398	623,732	15	2.40	312,375	30	9.60
Sirajganj	104,357	103,052	5	4.85	51,612	11	21.31
Sherpur	228,519	225,476	6	2.66	113,055	4	3.54
Narshingdi	204,319	201,641	12	5.95	101,004	12	11.88

^1^ Information on education missing for 0.01 (161) participants; ^2^ Information on occupation missing for 0.06 (746) participants.

**Table 3:** Suicide rates by age and sex, rural Bangladesh.

Age Group (Years)	Male				Female			
	PYO	Suicide (*n*)	Mortality Rate/100,000 PYO	Confidence Interval (CI)	PYO	Suicide (*n*)	Mortality Rate/100,000 PYO	Confidence Interval (CI)
<10	122,970	0	0.00		117,769	0	0.00	
10–14	72,339	4	5.53	1.51–14.16	69,510	1	1.44	0.04–8.02
15–17	33,156	3	9.05	1.87–26.44	28,815	4	13.88	3.78–35.54
18–24	57,909	3	5.18	1.07–15.14	75,339	7	9.29	3.74–19.14
25–64	236,636	9	3.80	1.74–7.22	269,712	5	1.85	0.60–4.33
65+	36,556	1	2.74	0.07–15.24	33,189	1	3.01	0.08–16.79
Total	559,566	20	3.57	2.18–5.52	594,335	18	3.03	1.79–4.79

**Table 4:** Unadjusted and adjusted analysis for fatal suicidal behavior by socio-demographic and geographical factors, rural Bangladesh.

Characteristics	Unadjusted		Adjusted	
	Incidence Rate Ratio (IRR)	95% CI	Incidence Rate Ratio (IRR)	95% CI
Age (in years)				
15–17	4.09 **	1.64–10.12	6.31 *	1.35–29.32
18–24	2.71 *	1.2–6.11	4.04 *	1.56–10.47
25–64	Reference		Reference	
65+	1.04	0.24–4.56	1.48	0.32–6.87
Sex				
Male	1.05	0.53–2.08	0.87	0.29–2.56
Female	Reference		Reference	
Education				
No education	2.81	0.36–21.75	5.48	0.56–53.10
Primary complete (five years)	1.50	0.17–12.83	2.27	0.22–22.62
Secondary complete (10 years)	4.15	0.54–31.25	4.04	0.49–32.97
Secondary and above	Reference		Reference	
Occupation				
Agriculture	1.40	0.33–5.84	0.99	0.22–4.45
Business	0.00		0.00	
Skilled labor	Reference		Reference	
Unskilled labor	1.25	0.13–12.01	1.20	0.12–11.82
Transport worker	5.14 *	1.03–25.48	4.25	0.82–21.82
Students	2.89	0.76–10.88	1.86	0.32–10.67
Retired/Unemployed/Housewife	0.95	0.27–3.33	0.62	0.13–2.87
Children (Under 12 years)	0.00		0.00	
Others (NA)	0.00		0.00	
Marital Status				
Married	0.68	0.31–1.47	3.60	0.92–14.06
Never-married	Reference		Reference	
Widowed/Divorced/Separated	0.00		0.00	
Wealth quintile				
Lowest	Reference		Reference	
Second	0.76	0.20–2.83	0.76	0.20–2.85
Middle	1.55	0.52–4.61	1.71	0.55–5.26
Fourth	1.44	0.48–4.28	1.61	0.51–5.07
Highest	0.92	0.28–3.01	1.03	0.28–3.71
District				
Chandpur/Comilla	Reference		Reference	
Sirajganj	2.48	0.87–7.02	2.32	0.77–6.88
Sherpur	1.19	0.41–3.37	0.97	0.33–2.89
Narshingdi	2.83 *	1.24–6.42	2.89 *	1.26–6.65

* *p* < 0.05; ** *p* < 0.01.

**Table 5:** Unadjusted and adjusted analysis of non-fatal suicidal behavior by socio-demographic and geographical factors, rural Bangladesh.

Characteristics	Unadjusted		Adjusted	
	Incidence Rate Ratio (IRR)	95% CI	Incidence Rate Ratio (IRR)	95% CI
Age (in years)				
15–17	0.97	0.34–2.72	1.01	0.25–4.03
18–24	1.46	0.77–2.77	1.31	0.59–2.91
25–64	Reference		Reference	
65+	0.44	0.10–0.82	0.49	0.11–2.18
Sex				
Male	1.07	0.63–1.84	0.63	0.24–1.67
Female	Reference		Reference	
Education				
No education	1.91	0.44–8.35	1.59	0.31–8.02
Primary complete (5 years)	2.23	0.51–9.75	1.60	0.45–2.00
Secondary complete (10 years)	2.71	0.64–11.55	2.28	0.66–3.03
Secondary and above	Reference			
Occupation				
Agriculture	1.15	0.46–2.87	1.49	0.56–3.97
Business	0.18	0.02–1.43	0.20	0.03–1.69
Skilled labor	Reference		Reference	
Unskilled labor	1.87	0.56–6.21	2.25	0.66–7.68
Transport worker	0.63	0.08–5.10	0.78	0.10–6.38
Students	0.41	0.10–1.54	0.21 *	0.04–1.00
Retired/Unemployed/Housewife	0.69	0.31–1.53	0.57	0.19–1.72
Children (Under 12 years)	0.00		0.00	
Others (NA)	0.00		0.00	
Marital Status				
Married	0.72	0.39–1.37	0.55	0.22–1.38
Never-married	Reference		Reference	
Widowed/Divorced/Separated	0.58	0.17–2.04	0.6	0.13–2.86
Children < 12 years	0.00			
Wealth quintile				
Lowest	Reference		Reference	
Second	1.27	0.53–3.00	1.17	0.49–2.80
Middle	1.15	0.48–2.71	1.12	0.46–2.71
Fourth	0.98	0.40–2.35	0.99	0.40–2.45
Highest	0.77	0.30–1.94	0.82	0.30–2.19
District				
Chandpur/Comilla	Reference		Reference	
Sirajganj	2.11 *	1.02–4.34	2.06	0.96–4.42
Sherpur	0.31	0.09–1.01	0.28 *	0.08–0.94
Narshingdi	1.34	0.68–2.63	1.34	0.67–2.66

* *p* < 0.05; ** *p* < 0.01.

## Supplementary Materials

The following are available online at www.mdpi.com/1660-4601/14/9/1032/s1, Table S1: Unadjusted and adjusted analysis of suicide among participants aged 10–17 year olds by socio-demographic and geographical factors, rural Bangladesh. Table S2: Unadjusted and adjusted analysis of attempted suicide among participants aged 10–17 year olds by socio-demographic and geographical factors, rural Bangladesh.

## Abbreviations

The following abbreviations are used in this manuscript:

LMIC	Low and Middle Income Countries
HIC	High Income Countries
CIPRB	Center for Injury Prevention and Research, Bangladesh
icddr,b	International Centre for Diarrhoeal Disease Research, Bangladesh (icddr,b)
JHSPH	John’s Hopkins School of Public Health
CI	Confidence Interval
IRR	Incidence Rate Ratio
PYO	Person Years Observed
