# Supplementary material for: The Burden of Suicide in Rural Bangladesh: Magnitude and Risk Factors
Source: Int J Environ Res Public Health. 2017 Sep 9;14(9):1032. doi: 10.3390/ijerph14091032 (PMC5615569; doi:10.3390/ijerph14091032)
Supplement: Supplementary file 1 [file ijerph-14-01032-s001.pdf]

Table S1. Unadjusted and adjusted analysis of suicide fatal suicidal behavior among participants aged 10-17 years by socio-demographic and geographical factors, rural Bangladesh

**Characteristics**	**Unadjusted**		**Adjusted**	
	IRR	95% CI	IRR	95% CI
Age (in years)				
10-14	Reference			
15-17	3.20*	1.02-10.10	2.23	0.46-10.90
Sex				
Male	1.3	0.41-4.11	3.69	0.87-15.57
Female	Reference		Reference	
Education				
No education	0.00	-		
Primary complete (5 years)	Reference		Reference	
Secondary complete (10 years)	1.70	0.54-5.35	0.79	0.18-3.55
Secondary and above	0.00	-		
Occupation				
Agriculture	0.00	-	0.00	-
Business	0.00	-	0.00	-
Skilled labor	0.00	-	0.00	-
Unskilled labor	0.00	-	0.00	-
Transport worker	0.00	-	0.00	-
Students	Reference		Reference	
Retired/unemployed/housewife	5.46**	1.64-18.13	1.63	0.29-9.12
Children (Under 12 years)	0.00	-	0.00	-
Others (NA)	0.00	-	0.00	-
Marital Status				
Married	20.18**	5.70-71.5	22.06**	3.70-131.63
Never M-married	Reference		Reference	
Widowed/Divorced/Separated	0.00	-	0.00	-
Children <12 years	0.78	0.16-3.86	0.94	0.14-6.39
Wealth quintile				
Lowest	Reference		Reference	
Second	0.41	0.04-4.49	0.36	0.03-6.39
Middle	0.76	0.11-5.37	0.60	0.08-4.36
Fourth	1.51	0.27-8.24	1.13	0.20-6.49
Highest	1.25	0.21-7.51	0.82	0.12-5.39
District				
Chandpur/Comilla	Reference		Reference	
Sirajganj	0.00	-	0.00	-
Sherpur	0.84	0.17-4.04	1.05	0.21-5.23
Narshingdi	1.32	0.34-5.12	1.28	0.33-5.01

*P<0.05; **p<0.01

Table S2. Unadjusted and adjusted analysis of attempted suicidenon-fatal suicidal behavior among participants aged 10-17 years by socio-demographic and geographical factors, rural Bangladesh

Characteristics	Unadjusted		Adjusted	
	IRR	95% CI	IRR	95% CI
Age (in years)				
10-14'	Reference			
15-17	2.29	0.57-9.15	1.21	0.27-9.36
Sex				
Male	1.54	0.37-6.43	1.22	0.27-5.54
Female	Reference		Reference	
Education				
No education	5.44	0.49-60.0	3.51	0.26-48.08
Primary complete (5 years)	Reference		Reference	
Secondary complete (10 years)	3.03	0.59-15.64	6.73	0.89-50.87
Secondary and above	0.00	-	0.00	-
Occupation				
Agriculture	0.00	-	0.00	-
Business	0.00	-	0.00	-
Skilled labor	5.01	0.59-42.0	5.32	0.49-57.26
Unskilled labor	11.41*	1.33-97.64	15.75*	1.35-184.52
Transport worker	0.00	-	0.00	-
Students	Reference		Reference	
Retired/unemployed/housewife	2.19	0.26-18.7	2.53	0.24-26.40
Children (Under 12 years)	0.00	-	0.00	-
Others (NA)	0.00	-	0.00	-
Marital Status				
Married	0.00	-	0.00	-
Never M-married	Reference		Reference	
Widowed/Divorced/Separated	0.00	-	0.00	-
Children <12 years	0.78	0.16-3.86	3.89	0.42-36.33
Wealth quintile				
Lowest	Reference		Reference	
Second	2.45	0.25-23.56	2.52	0.25-25.03
Middle	2.27	0.24-21.86	2.31	0.23-23.70
Fourth	0.76	0.04-12.08	0.78	0.05-13.37
Highest	0.00	-	0.00	-
District				
Chandpur/Comilla	Reference		Reference	
Sirajganj	1.4	0.15-11.64	1.27	0.14-11.40
Sherpur	0.6	0.07-5.06	0.48	0.05-4.34
Narshingdi	0.6	0.07-5.31	0.74	0.09-6.39

*P<0.05; **p<0.01
